# Teaching Real-World Evidence: Protocol for a Systematic Review

**DOI:** 10.2196/16941

**Published:** 2020-01-30

**Authors:** Ching Lam, Michelle Helena van Velthoven, Hassan Chaudhury, Edward Meinert

**Affiliations:** 1 Digitally Enabled Preventative Health Research Group Department of Paediatrics University of Oxford Oxford United Kingdom; 2 Department of Primary Care and Public Health Imperial College London London United Kingdom

**Keywords:** real-world evidence, teaching, public health

## Abstract

**Background:**

Real-world evidence (RWE) refers to observational health care data beyond clinical trial data. It holds the promise of transforming health care as a new form of evidence to support decision makers in making decisions when developing and regulating medicines. As the importance of RWE is recognized by industry and regulatory bodies, teaching RWE becomes an important matter to evaluate and refine in order to develop future researchers and stakeholders who can better integrate RWE into the routine development of medicine.

**Objective:**

The aim of this review is to understand how RWE is currently being taught. From this landscape study, the insufficiencies of the current education of RWE can be identified and subsequently inform future education policies around RWE and its subfacets.

**Methods:**

We will search MEDLINE, EMBASE, PsycINFO, Healthcare Management Information Consortium, Cochrane, and Web of Science for published studies using a combination of keywords and subject headings related to RWE and education. In addition, a Google search to identify grey literature will be conducted. Two authors will independently screen the titles and abstracts identified from the search and accept or reject the studies according to the study inclusion criteria; any discrepancies will be discussed and resolved. The quality of the included literature will be assessed using the Critical Appraisal Skills Programme systematic review checklist.

**Results:**

Data from eligible publications will be abstracted into a predesigned form in order to better understand the current state of education of RWE and inform future RWE education directions and policies.

**Conclusions:**

The subsequent systematic review will be published in a peer-reviewed journal.

**International Registered Report Identifier (IRRID):**

PRR1-10.2196/16941

## Introduction

Real-world evidence (RWE) is a subset of evidence-based medicine that refers to health care information gathered through means outside of the typical clinical research settings. Evidence-based medicine (EBM) is the use of best available clinical evidence from systematic research combined with clinical expertise to deliver the best possible clinical care to patients [1,2]. Since its advent in the 1990s, EBM has been shown to be the cornerstone of the medical profession, raising awareness of using reliable, published evidence to aid decision making in medicine [3,4]. Sources of RWE data can come from electronic health records (EHRs), health surveys, claims and billing data, product and disease registries, mobile health apps, and personal smart devices. Collectively, the real RWE base can generate invaluable insights and findings on diseases, products, and patient populations [5,6].

Regulatory bodies around the world are gaining interest in RWE. Notably, the US Food and Drug Administration has recently announced a $100 million project to build a modern system that will gather RWE from approximately 10 million individuals [7], and the European Union has funded over 170 initiatives related to RWE, of which 65 received over 734 million euros of public funding [8]. According to a report from McKinsey and Company [9], pharmaceutical companies have recognized the impact of RWE and are actively applying it to safety, postmarket, and end-to-end product development to facilitate research and development and commercial and safety decisions.

The anticipated importance of data collection and analysis of RWE in the future of clinical trials and development of medicines is evident. In order to meet the future demands of RWE researchers in terms of realizing the true potential of RWE in transforming health care, it is important to educate stakeholders such as researchers, clinicians, and policy makers in RWE in an appropriate way.

EBM has long been taught to medical professionals, including clinicians and nurses [10-12], and due to its importance in clinical care, teaching EBM in medical school has been investigated in various studies. Smith et al [13] conducted a controlled trial to look into the effectiveness of EBM courses for residents. Another recent study conducted by Nasr et al [14] evaluated four EBM workshops taught to residents-in-training and postgraduates in medical school. Interestingly, Slawson et al [15] suggested the importance of information management in the teaching of EBM back in 2005, and with the rapid development of big data, better computer processors, and the maturation of machine learning in recent years, information management is more important than ever in EBM. However, it is unknown how RWE is being taught and what the effects are.

This systematic review aims to answer the following research questions:

- What are the current methods used to teach RWE, and what are the effects of those methods?
- Who are the stakeholders teaching and learning about RWE?

## Methods

### Review Conduct

This systematic review will be conducted following the Preferred Reporting Items for Systematic Reviews and Meta-Analyses (PRISMA) guidelines [16]. The protocol methods will be reported following the 2015 PRISMA checklist [17]. The protocol will be registered with PROSPERO.

### Eligibility Criteria

#### Inclusion Criteria

In accordance with PRISMA Protocols (PRISMA-P) checklist recommendation, the inclusion criteria for this protocol are in accordance with participants, interventions, comparators, and outcomes (PICO). Details of PICO to be included in the review are described in Textbox 1.

**Participants, interventions, comparators, and outcomes for review inclusion criteria.**

Population:

- Participants with real-world evidence teaching

Intervention:

- Any form of real-world evidence teaching

Comparator:

- No real-world evidence teaching

Outcomes:

- Learner-focused outcomes such as attitudes, cognitive changes, learner satisfaction

Study type:

- Any study type (study type will not be subjected to any restrictions)
- Must describe relevance to real-world evidence and its application in health care
- Must describe a teaching method
- English-language publications

#### Exclusion Criteria

Education methods that do not describe their relevance to RWE and its applications to health care will be excluded to limit the scope of the review to RWE-focused education and courses. Websites and articles describing RWE (although helpful for those actively seeking to learn more about RWE) will be excluded as there are no methods of teaching included. Studies not published in English are excluded due to the language barrier.

### Search Strategy

The following databases will be searched: MEDLINE, EMBASE, PsycINFO, Healthcare Management Information Consortium, Cochrane, and Web of Science. In addition, a Google search for grey literature such as blog posts, opinion pieces, press releases, and online courses will be conducted. Online course platforms such as Coursera, edX, FutureLearn, and OpenClassrooms will be searched to identify relevant courses. Textbox 2 shows the search concept and keywords to be searched for this review. Search strings will be constructed using a combination of RWE-related and education-related keywords.

**Concepts and keywords for search term development.**

Real-world evidence:

- Real world evidence, RWE, big data analytics, real world data, electronic health record*

Teaching:

- Medical education, medical student*, medical curriculum, medical school*, health professionals

### Study Selection

EndNote X8 software (Clarivate Analytics) will be used for the removal of duplicates. Textbox 1 describes the inclusion criteria of the review. Two independent reviewers will screen the titles and abstracts of papers to minimize the risk of bias and the risk of not including eligible papers due to oversight. Papers that are ineligible will be eliminated, and the full text of those that appear to meet the review’s eligibility criteria will be obtained and read in full to ensure eligibility. Any contradictions or discrepancies between the reviewers that arise will be discussed until consensus is reached. Valid studies will be assessed for their quality before any extraction of information.

### Quality Assessment and Risk of Bias

Two reviewers will independently check each article to minimize bias using the Cochrane Collaboration’s risk of bias tool as described in the Cochrane Handbook for Systematic Review of Interventions [18]. All included articles will be judged for their quality based on the Critical Appraisal Skills Programme systematic review checklist [19] and data analysis.

### Data Extraction

**Figure 1:**
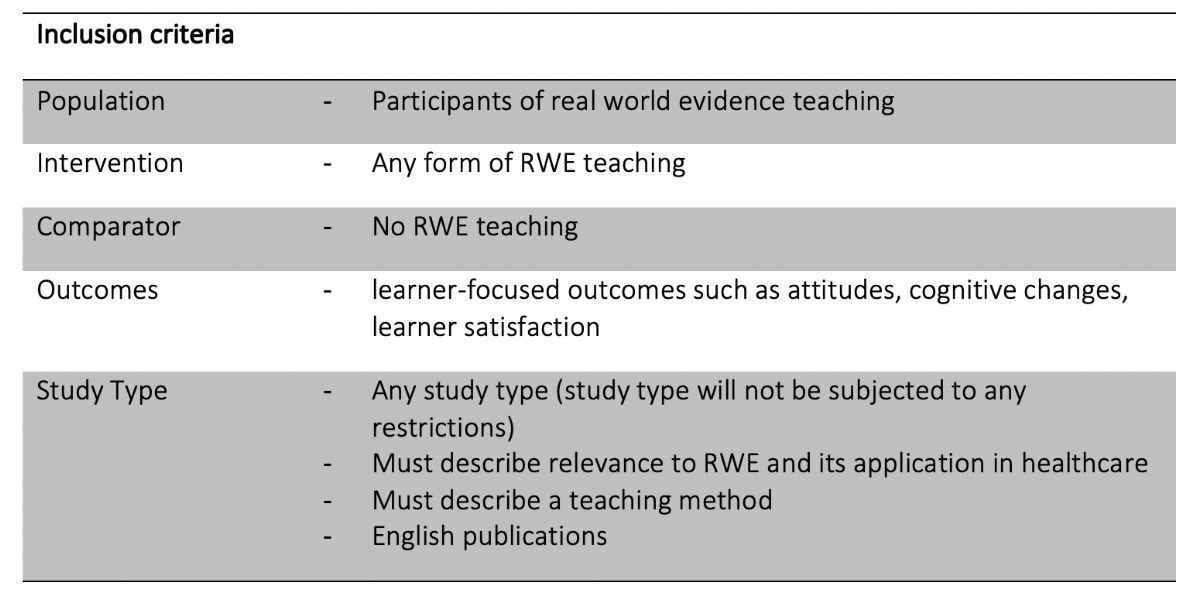
Sample data abstraction form.

Eligible sources will subsequently be reviewed in detail, and key relevant challenges will be extracted, categorized, and recorded into a predesigned Excel spreadsheet (Microsoft Corp). A sample data abstraction form can be found in Figure 1.

## Results

A sample search was conducted using PubMed, and the sample search string returned 943 results: (“real world evidence” OR “RWE” OR “big data analytic*” OR “real world data” OR “electronic health record*”) AND (“medical education” OR “medical student*” OR “medical curriculum” OR “medical school*”). The search string will be further fine-tuned in the review.

## Discussion

### Principal Findings

This study will offer a comprehensive overview of how RWE is taught to different stakeholders of the research and application of RWE in health care. However, traditional means of teaching, such as university lecturies, may not be published and hence may be underrepresented in this protocol.

### Conclusions

This protocol will be executed in 2020 and published in a peer-reviewed journal in accordance with PRISMA guidelines. Any deviations in the execution shall be noted in the subsequent systematic review publication. The findings from this review will be used to inform the education strategy of RWE.

## Abbreviations

EBM: evidence-based medicine
EHR: electronic health record
PICO: participants, interventions, comparators, and outcomes
PRISMA: Preferred Reporting Items for Systematic Reviews and Meta-Analyses
PRISMA-P: Preferred Reporting Items for Systematic Reviews and Meta-Analyses Protocols
RWE: real-world evidence
